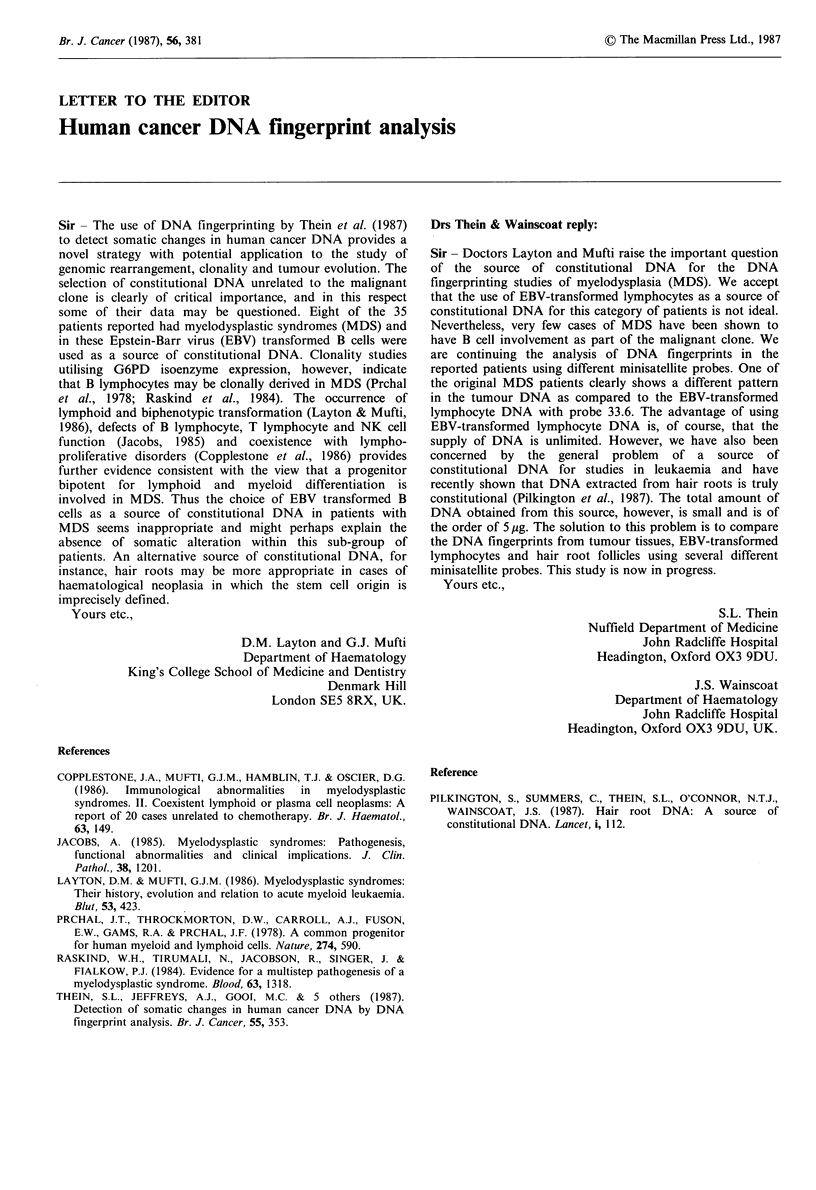# Drs Thein & Wainscoat reply

**Published:** 1987-09

**Authors:** S.L. Thein, J.S. Wainscoat


Yours etc.,

D.M. Layton and G.J. Mufti

Department of Haematology

**Drs Thein & Wainscoat reply:**

**Sir** – Doctors Layton and Mufti raise the important question of the source of constitutional DNA for the DNA fingerprinting studies of myelodysplasia (MDS). We accept that the use of EBV-transformed lymphocytes as a source of constitutional DNA for this category of patients is not ideal. Nevertheless, very few cases of MDS have been shown to have B cell involvement as part of the malignant clone. We are continuing the analysis of DNA fingerprints in the reported patients using different minisatellite probes. One of the original MDS patients clearly shows a different pattern in the tumour DNA as compared to the EBV-transformed lymphocyte DNA with probe 33.6. The advantage of using EBV-transformed lymphocyte DNA is, of course, that the supply of DNA is unlimited. However, we have also been concerned by the general problem of a source of constitutional DNA for studies in leukaemia and have recently shown that DNA extracted from hair roots is truly constitutional (Pilkington *et al.*, 1987). The total amount of DNA obtained from this source, however, is small and is of the order of 5 $\mu$g. The solution to this problem is to compare the DNA fingerprints from tumour tissues, EBV-transformed lymphocytes and hair root follicles using several different minisatellite probes. This study is now in progress.

Yours etc.,

S.L. Thein
Nuffield Department of Medicine
John Radcliffe Hospital
Headington, Oxford OX3 9DU.

J.S. Wainscoat
Department of Haematology
John Radcliffe Hospital
Headington, Oxford OX3 9DU, UK.

**Reference**

PILKINGTON, S., SUMMERS, C., THEIN, S.L., O'CONNOR, N.T.J., WAINSCOAT, J.S. (1987). Hair root DNA: A source of constitutional DNA. *Lancet*, **i**, 112.